# Effects of sleep quality on diurnal volumetric change of brain in older adults

**DOI:** 10.1002/alz.088907

**Published:** 2025-01-09

**Authors:** Jun Sung Kim, Ji Won Han, Dae Jong Oh, Seung Wan Suh, Sungman Jo, Min Jeong Kwon, Jieun Park, Jae Hyoung Kim, Ki Woong Kim

**Affiliations:** ^1^ Seoul National University Bundang Hospital, Seongnam Korea, Republic of (South); ^2^ Seoul National University College of Medicine, Seoul Korea, Republic of (South); ^3^ SMG‐SNU Boramae Medical Center, Seoul Korea, Republic of (South); ^4^ Kangdong Sacred Heart Hospital, Hallym University College of Medicine, Seoul Korea, Republic of (South); ^5^ Seoul National University, Seoul Korea, Republic of (South); ^6^ Seoul National University College of Natural Sciences, Seoul Korea, Republic of (South); ^7^ Seoul National University College of Natural Science, Seoul Korea, Republic of (South); ^8^ Seoul National University College of Medicine, Seongnam Korea, Republic of (South); ^9^ College of Medicine, Seoul National University, Seoul Korea, Republic of (South)

## Abstract

**Background:**

Brain volume is influenced by several factors that can change throughout the day. In addition, most of these factors are influenced by sleep quality. This study investigated diurnal variation in brain volume and its relation to overnight sleep quality.

**Method:**

We enrolled 1,003 healthy Koreans without any psychiatric disorders aged 60 years or older. We assessed sleep quality and average wake time using the Pittsburgh Sleep Quality Index, and divided sleep quality into good, moderate, and poor groups. We estimated the whole and regional brain volumes from three‐dimensional T1‐weighted brain MRI scans. We divided the interval between average wake‐up time and MRI acquisition time (INT) into tertile groups: short (INT_1_), medium (INT_2_), and long (INT_3_).

**Result:**

Whole and regional brain volumes showed no significance with respect to INT. However, the interaction between INT and sleep quality showed significance for whole brain, cerebral gray matter, and cerebrospinal fluid volumes (p < .05). The INT_2_ group showed significantly lower volumes of whole brain, whole gray matter, cerebral gray matter, cortical gray matter, subcortical gray matter, and cerebrospinal fluid than the INT_1_ and INT_3_ groups only in the individuals with good sleep quality.

**Conclusion:**

Human brain volume changes significantly within a day associated with overnight sleep in the individuals with good sleep quality.

